# Evaluation of a prediction model for colorectal cancer: retrospective analysis of 2.5 million patient records

**DOI:** 10.1002/cam4.1183

**Published:** 2017-09-21

**Authors:** Jacqueline Birks, Clare Bankhead, Tim A. Holt, Alice Fuller, Julietta Patnick

**Affiliations:** ^1^ Oxford Biomedical Research Centre Oxford University Botnar Research Institute Windmill Rd Oxford OX7 3LD United Kingdom; ^2^ Nuffield Department of Primary Care Health Sciences Oxford University Radcliffe Observatory Quarter Woodstock Road Oxford OX2 6GG United Kingdom; ^3^ Cancer Epidemiology Unit Nuffield Department of Population Health Oxford University Richard Doll Building, Old Road Campus Oxford OX3 7LF United Kingdom

**Keywords:** Blood cell count, colorectal neoplasms, early detection of cancer, electronic health records, machine learning, risk assessment

## Abstract

Earlier detection of colorectal cancer greatly improves prognosis, largely through surgical excision of neoplastic polyps. These include benign adenomas which can transform over time to malignant adenocarcinomas. This progression may be associated with changes in full blood count indices. An existing risk algorithm derived in Israel stratifies individuals according to colorectal cancer risk using full blood count data, but has not been validated in the UK. We undertook a retrospective analysis using the Clinical Practice Research Datalink. Patients aged over 40 with full blood count data were risk‐stratified and followed up for a diagnosis of colorectal cancer over a range of time intervals. The primary outcome was the area under the receiver operating characteristic curve for the 18–24‐month interval. We also undertook a case–control analysis (matching for age, sex, and year of risk score), and a cohort study of patients undergoing full blood count testing during 2012, to estimate predictive values. We included 2,550,119 patients. The area under the curve for the 18–24‐month interval was 0.776 [95% confidence interval (CI): 0.771, 0.781]. Performance improves as the time interval reduces. The area under the curve for the age‐matched case–control analysis was 0.583 [0.574, 0.591]. For the population risk‐scored in 2012, the positive predictive value at 99.5% specificity was 8.8% with negative predictive value 99.6%. The algorithm offers an additional means of identifying risk of colorectal cancer, and could support other approaches to early detection, including screening and active case finding.

## Background

Colorectal cancer is the fourth commonest cancer in the UK with 41,265 new cases in 2014, representing 11% of all new cancer 1. Incidence is strongly related to age, with 72% of cases (2012–2014) occurring in those older than 65 years 2. Five‐year survival is heavily influenced by stage at diagnosis, and is over 95% at stage I but less than 8% at stage 4 3. Because the symptoms develop insidiously, a high proportion of patients are diagnosed at a stage beyond surgical cure.

Most cases develop slowly over a number of years from benign adenomatous polyps which transform to malignant adenocarcinomas. The identification of the early stages offers the opportunity to improve outcomes through surgical excision 4. Adenomas and adenocarcinomas may bleed to a level unnoticed by the individual, enabling detection through fecal occult blood testing (FOBt), and this has become the standard screening approach in the UK 5. A meta‐analysis published in 2007 found a 16% reduction in colorectal cancer‐specific mortality associated with FOBt screening (odds ratio 0.84, 95% CI: 0.78, 0.89) 6. All countries in the UK now offer FOBt for some age ranges of the older population, at two yearly intervals. However, uptake of screening invitations was only 57% in randomized trials, and a pilot study of the FOBt in England reported a 59% response 7. These rates are clearly suboptimal and additional approaches might facilitate early detection in a greater proportion of the population.

Routinely collected primary care data have been used to develop a range of algorithms for identifying individuals at risk of various types of cancers 8, 9, including colorectal 10, 11, 12. These algorithms use the presence of symptoms recorded in primary care to identify individuals with a high risk of being later diagnosed. The limitation is that patients so identified already have established symptoms noticeable enough to report to a doctor, and are therefore at a relatively late stage.

Medial EarlySign, Israel, is developing a range of machine learning algorithmic tools to identify high‐risk patients in various settings (diabetes control, cardiovascular procedures, birth‐related complications, deterioration in intensive care units, cancer screening, genetic screening). The algorithms are developed by analyzing large complex datasets of millions of patients. Recently, their group reported a study on an Israeli population of two million patients 13. They analyzed primary care electronic health record data to identify individuals diagnosed with colorectal cancer. A machine learning process derived a prediction algorithm based on a random forest model. Variables included age, sex, and full blood count (FBC) results, which are commonly found in primary care health records. The rationale is that patients may develop subtle changes in FBC indices at a relatively early stage, prior to the development of overt symptoms. This may enable earlier detection than is possible through the symptom‐based approach, and prior to the onset of anemia. Hamilton et al. demonstrated that anemia evident in full blood count data from UK primary care predicts colorectal cancer risk, and that iron deficiency, which may predate established anemia, is an independent risk factor 14. Signs of iron deficiency may develop in full blood count profiles despite hemoglobin remaining in the reference range. If more than one FBC result is available, the algorithm can detect changes in the values of the indices, identifying a problem even before they are out of their reference ranges. Such changes are unlikely to be noticed by a clinician examining FBC reports, which may be filed unseen if all indices are in range.

The Israeli model was trained on a derivation dataset and then tested on validation datasets, one from the same source, the other from a sample of UK data from The Health Improvement Network (THIN) 13. Several measures were used to evaluate performance, including the area under the receiver operating characteristic curve, or “area under the curve” (AUC), and statistics related to the AUC, where the threshold of the risk score was set to specific values of sensitivity (50%) and specificity (99.5%). The algorithm's performance was assessed at different time intervals up to 24 months before the diagnosis on individuals aged 50–75 years. For the interval 3–6 months before diagnosis, the specificity was 88(± 2)% at a sensitivity of 50% and the AUC was 0.82. A sensitivity of 50% is similar to that offered by FOBt, that is, 50% of cancers will give a positive result 15.

The aim of this current study is to evaluate this risk algorithm independently using Clinical Practice Research Datalink (CPRD) data from the UK.

## Methods

### Study design, source of data, and study population

We undertook a retrospective analysis, following the methods described in Kinar et al., in a population of patients from the CPRD, a large database linked to anonymized patient‐level data from over 600 UK general practices. Study participants were those registered on the database between 01 January 2000 and 28 April 2015**.** The end date was the date of the most recent update of the CPRD dataset, which may differ between contributing practices. All patients included in the study had at least one FBC present in the record. Entry to the cohort was at the index date, which is defined as the latest of start date, the 40th birthday, or date of registration with the general practice.

We excluded the following groups:

- Less than 12 months registered with the general practice
- Less than 2 years of follow‐up data following the index date
- History of colorectal cancer before the index date
- Colorectal cancer precursors (e.g., adenomatous polyps)
- Hemoglobin gene defects (thalassemia, sickle cell disorders)

We included all other patient records, to maximize the precision of our estimates.

### Study outcome

The primary endpoint is a first diagnosis of colorectal cancer in the primary care record. Patients with no diagnosis were censored at the earliest date of: date of death, the end date or date of leaving the general practice.

### Predictors

We extracted the year of birth and sex of each participant, every FBC, each of which could have a maximum of 20 indices, and the date of each FBC.

### Statistical methods

The calculation of the risk score uses the patient identity code, year of birth, sex, and date of FBC. The algorithm requires up to 20 indices of a single FBC, which are imputed if missing. Previous values for the FBC indices are utilized if available but only one FBC is required to generate a risk score. Input files were prepared by the Oxford team, excluding the outcome data. A representative from the Medial team ran the algorithm and returned a dataset containing a score, between 0 and 100, for every FBC considered valid according to their criteria, together with the date of the FBC and the patient identity code.

For the primary analysis, we followed the methods outlined in the paper reporting the original derivation and validation of the score by *Kinar* et al. We identified the FBCs at least 18 months prior to the end date for those with no diagnosis (noncases) of colorectal cancer. For each patient, one FBC and associated score was randomly selected using a computer algorithm. For those with a diagnosis (cases), all FBCs within 18–24 months prior to date of diagnosis were identified. If a patient had more than one FBC within the time period, one FBC was randomly selected using a computer algorithm. Logistic regression analysis was performed with the score as the only predictor in the model. To evaluate the performance of the model, the receiver operating characteristic (ROC) curve was plotted and the area under the curve (AUC) calculated (C‐statistic). To provide statistics which could be compared to those reported by *Kinar* et al., the specificity was calculated when sensitivity was 50%, and sensitivity calculated when specificity was 99.5%.

In contrast to the *Kinar* et al. study, whose primary analysis was based on the 3–6‐month time interval prior to diagnosis, we chose 18–24 months for our primary analysis. This was because the opportunity to modify prognosis requires an adequate time interval for intervention. For our secondary analyses, the performance of the algorithm was measured at 3–6, 6–12, 12–18, and 24–36 months before diagnosis using the same method.

### Sensitivity analyses

To test whether the outcomes were robust to variation in the randomly selected FBCs, the analysis was repeated 10 times, resampling the dataset using a different seed code.

As a further sensitivity analysis, a case–control study was designed by matching cases and noncases by sex, year of birth, and year of selected FBC. This was undertaken because of the (expected) difference in the age distributions of the cases and noncases populations.

For those with no diagnosis (noncases) we identified the FBCs at least 18 months prior to the end date. For those with a diagnosis (cases), all FBCs within 18–24 months prior to date of diagnosis were identified. If a patient, cases and noncases, had more than one FBC within the time period, one FBC was randomly selected. For each case 100 noncases were selected matching for sex, age at time of FBC, and year of risk score. Logistic regression analysis was performed with the score as the only predictor in the model. The AUC for the receiver operating characteristic (ROC) curve was calculated (C‐statistic).

### Calculation of predictive values

For the analyses set out above, estimation of predictive values (PPV, NPV) is not appropriate, as there is no defined population to which they would be applicable. It is possible to define a cohort of patients at the start date of the study, 01 January 2000, but the concept of a well‐defined cohort is lost as each patient contributes data at a date set by the randomly selected score. To investigate the result of applying the score to a defined cohort of patients presenting in primary care, we followed until end date all patients with a FBC and score in 2012 who had not previously been diagnosed with colorectal cancer. The year 2012 was chosen as this was the most recent year for which 24 months of follow‐up were available.

All patients were followed until they were either diagnosed with colorectal cancer or censored at end date. We conducted a logistic regression analysis with a diagnosis within 24 months as the outcome and the risk score as predictor. Patients who were censored within 2 years of the score in 2012, either because they had died without a diagnosis or were lost to follow‐up, were omitted from the analysis. The AUC for the ROC curve was calculated (C‐statistic). We also calculated values for specificity when sensitivity was 50%, sensitivity when specificity was 99.5%, positive predictive value (PPV), and negative predictive value (NPV).

We could not assess agreement between observed outcomes and predicted outcomes, that is calibration, as the risk score does not provide a measure of absolute risk.

The project was approved by the Independent Scientific Advisory Committee (ISAC) for CPRD (protocol 14_195RMn) and the protocol was made available to the reviewers.

## Results

We identified 2,914,589 patients who met the inclusion criteria and had at least one FBC. The mean number of indices comprising a FBC was 11.8. The percentage count of neutrophils, monocytes, eosinophils, basophils, and lymphocytes (components of white blood cells) were recorded for only 10% of FBCs but these can be derived from the cell counts. The mean cell volume, mean cell hemoglobin and mean cell hemoglobin concentration can also be derived from values of other indices. This increased the mean number of indices available to 15.6. Those most frequently missing were mean platelet volume (present in 14% of FBCs) and red cell distribution width (present in 2% of FBCs). The Table S1 gives the proportions of missing data for each FBC parameter.

Data were excluded when more than one FBC belonging to an individual patient was associated with a single date or when hemoglobin was missing from the FBC. A total of 364,470 patients were excluded as they had insufficient FBC indices to generate a score. The total number of patient years of data from the remaining 2,550,119 patients was 27,949,304 indicating that the average time in the study was 11.0 years. The mean age at the start date was 54.2 years, and 55.4% of the patients were female.

For the included patients, we identified 25,430 with an incident diagnosis of colorectal cancer. We excluded 68 patients whose age at diagnosis was less than 40 years. The mean age of diagnosis was 71.2 years. 20,492 cases had at least one FBC before diagnosis. The time of the closest FBC to diagnosis varied between 0 and 5580 days. A total of 7634 cases had a FBC within 2 years before diagnosis.

### Primary analysis

We identified 2,220,108 patients without a diagnosis who had at least one FBC and related score at least 18 months before end date, and 5141 patients with a diagnosis who had at least one FBC and related score within the time window of 18 to 24 months before diagnosis. For each patient that met the inclusion criteria, one FBC and associated score was randomly selected. The mean age (standard deviation) of those without a diagnosis was 60.5 years (14.0) and of those with a diagnosis, 72.7 years (10.5). The mean age at time of the FBC and the mean score by sex and colorectal cancer diagnosis are reported in Table 1.

**Table 1:** Description of the population included in the primary analysis

	No diagnosis of CRC			Diagnosis of CRC		
	Number	Age (SD) range	Score (SD) range	Number	Age (SD) range	Score (SD) range
Female	1240666	60.8 (14.7) 40–108	48.3 (28.7) 0–100	2410	73.4 (11.0) 40–98	75.4 (21.1) 0–100
Male	979442	60.2 (13.2) 40–110	55.6 (29.0) 0–100	2731	72.1 (9.9) 40–97	82.5 (17.3) 6–100
Total	2220108	60.5 (14.0) 40–110	51.5 (29.0) 0–100	5141	72.7 (10.5) 40–98	79.1 (19.5) 0–100

**Figure 1:**
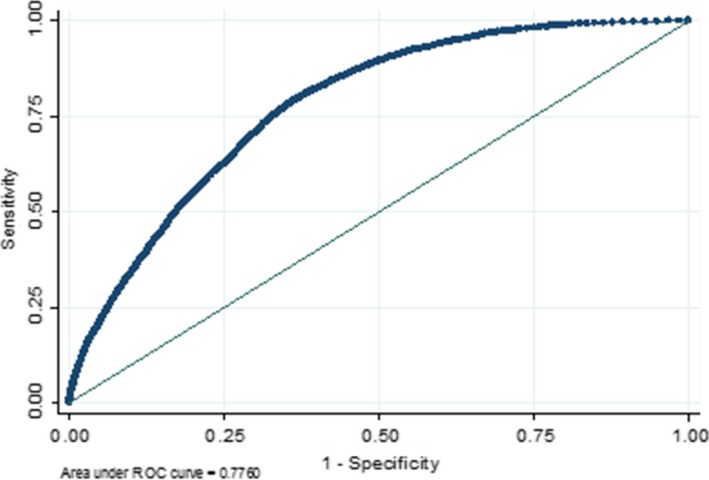
The receiver operating characteristic curve for the 18–24‐months' time interval (primary analysis).

The results of the logistic regression analysis with the score as the only predictor are reported in Table 2 (shaded row). The ROC curve is shown in Figure 1 and gives an AUC of 0.776 [95% CI: 0.771, 0.781].

**Table 2:** Results of logistic analysis for a diagnosis of colorectal cancer with score as the predictor, at five time intervals before diagnosis

Time before diagnosis (months)	Total number	Cases	Noncases	AUROC (95% CI)	Specificity when sensitivity = 50%	Sensitivity when specificity = 99.5%
3–6 m	2484699	5935	2478764	0.844 (0.839, 0.849)	92.50 (92.46, 92.53)	14.2 (13.3, 15.1)
					Score cut‐off = 96.16	Score cut‐off = 99.82
6–12 m	2436324	6821	2429503	0.813 (0.809, 0.818)	86.98 (86.94, 87.02)	9.3 (8.6, 10.0)
					Score cut‐off = 89.63	Score cut‐off = 99.81
12–24 m	2334380	5744	2328636	0.791 (0.786, 0.796)	84.98 (84.94, 85.03)	6.2 (5.6, 6.9)
					Score cut‐off = 86.04	Score cut‐off = 99.79
18–24 m	2225249	5141	2220108	0.776 (0.771, 0.781)	82.73 (82.68, 82.78)	3.91 (3.40, 4.48)
					Score cut‐off = 83.47	Score cut‐off = 99.78
24–36 m	2110307	7360	2102947	0.751 (0.746, 0.756)	79.41 (79.36, 79.47)	2.5 (2.2, 2.9)
					Score cut‐off = 80.22	Score cut‐off = 99.77

The primary analysis was for the 18–24‐month interval (shaded).

### Secondary analyses

Table 2 also gives the results of repeating the analysis with different time windows. The closer to the diagnosis, the greater become the sensitivity, specificity, and AUC values.

### Sensitivity analyses

The resampling exercise confirmed that the analyses were robust to variation in the randomly selected seed codes. The primary outcome AUC gave a narrow range of values in the 10 samples (0.7755 to 0.7765) around a mean of 0.7760. The risk score cut off associated with 99.5% specificity was identical (99.78) for all ten.

Tables 3 reports the results of the case–control study matching cases and noncases by sex, year of birth, and year of selected FBC. The AUC was 0.583 [0.574, 0.591].

**Table 3:** Results of the case–control sensitivity analysis

Descriptive statistics						
	No diagnosis of CRC			Diagnosis of CRC		
	Number	Age (SD) Range	Score (SD) Range	Number	Age (SD) Range	Score (SD) Range
Female	291273	73.5 (10.7) 40‐98	71.5 (20.0)0–100	2410	73.4 (11.0) 40–98	75.4 (21.1)0–100
Male	222827	71.6 (10.1) 40‐98	78.6 (18.4)2–100	2731	72.1 (9.9) 40–97	82.5 (17.3)6–100
Total	514100	71.6 (10.1) 40‐98	78.6 (18.4)2–100	5141	72.7 (10.5) 40–98	79.1 (19.5)0–100

Statistics of discrimination					
Total number	Cases	Noncases	AUROC (95% CI)	Specificity when sensitivity = 50%	Sensitivity when specificity = 99.5%
519241	5141	514100	0.583 (0.574, 0.591)	63.2 (63.0, 63.3)	2.0 (1.7, 2.4)
				Score cut‐off = 83.69	Score cut‐off = 99.88

For the estimation of predictive values, we identified 797,119 patients with a score measured in 2012, and with no diagnosis of colorectal cancer at the time of this score. Male and female patients were pooled, age range 40‐108 years. Patients without 2 years of follow‐up from score were excluded. Within 2 years 36,032 (4.5%) of the patients had died and 160,814 (20%) were lost to follow‐up. The mean age and score of these groups are reported in Table 4. The analysis dataset consists of the patients with a diagnosis within 2 years of the score, 2454 patients, and those with 2 years of follow‐up without a diagnosis (594,926 patients). The results of the logistic regression analysis with the score as the only predictor are reported in Table 5.

**Table 4:** Description of patients included in the cohort who had a score measured in 2012 and with follow‐up for 24 months

	Number	Mean age at 2012 (SD)	Mean score (SD)
Patients lost to follow‐up within 2 years without a diagnosis	160814	63.5 (14.1)	58.1 (28.0)
Patients who had died within 2 years without a diagnosis	36032	79.0 (11.6)	86.4 (15.9)
Patients in analysis set	600273	62.9 (13.5)	56.8 (27.6)
Total	797119	63.8 (13.9)	58.4 (28.0)

**Table 5:** Results of the 2012 cohort analysis. The threshold for case identification is a risk score cut off of 99.84 (associated with 99.5% specificity)

Total number	Cases of CRC	Noncases	True positive	False positive	False negative	True negative	AUROC (95% CI)	Sensitivity when specificity=99.5%
600273	2454	597819	280	2893	2174	594926	0.781 (0.772, 0.791)	11.4 (10.2, 12.7)
								Score cut‐off = 99.84

Assuming that the patients included in the analysis reported in Table 4 represent a defined cohort of patients, the positive predictive value (PPV) and negative predictive value (NPV) were estimated using a cut off for the score of 99.84 (associated with 99.5% specificity). The PPV was 8.8% with NPV 99.6%. At this cut off, the odds ratio for a diagnosis of colorectal cancer was 26.5 (95% CI: 23.3, 30.2). This figure is very similar to the value reported by *Kinar* et al.

## Discussion

### Summary of main findings

The risk score applied to routinely collected primary care data from the UK produced AUC values comparable with those from the Israeli population used to derive it. Our primary outcome interval (18–24 months) produced an AUC of 0.776. This interval was chosen as it provides a greater opportunity to intervene and modify prognosis than the 3–6‐month interval chosen by *Kinar* et al. for their primary analysis, whose AUC was 0.82. In our study, this shorter time interval gave a very similar value of 0.84, validating the risk algorithm performance in the UK. Most of the predictive power is due to age, as is evidenced by the reduction in AUC to 0.583 in the case–control sensitivity analysis, which removes the age component through age matching. However, age is indeed a useful factor in determining colorectal cancer risk, and the addition of FBC indices to age and sex improves the ability to identify patients at risk.

### Limitations of the study

After removing our excluded groups, our primary analysis only included 5141 of 25,430 colorectal cancer patients in the dataset. The FBC data from the Israeli population consist of more indices than are found in the UK CPRD data. Patients in Israel have full blood counts taken routinely as part of regular health checks, while in the UK, the test is usually conducted for some clinical reason. The fact that a blood test has been requested therefore carries some predictive weight itself in the UK setting, although FBCs are requested for a wide variety of reasons, most unrelated to colorectal cancer, and our results were similar to those found by *Kinar* et al. in the Israeli setting.

Other known risk factors for colorectal cancer, such as family history and microsatellite instability (MSI) status are not included in this approach. MSI status is not usually available in the primary care setting, and family history is recorded inconsistently. One of the advantages of this risk algorithm is that provided an individual has had a full blood count taken, the values of all the indices should be available, with very little missing data.

Absolute risk is assumed to be a monotonic function of risk score, but the form of the function is unknown. Discrimination can be investigated using sensitivity, specificity, and the area under the ROC curve, but we cannot easily quantify how close the predictions of risk are to actual risk.

The outcomes recorded in CPRD and used in this study are incident diagnoses of colorectal cancer following the risk estimation, which is different from the prevalence of undiagnosed cancer in those identified. This prevalence could only be measured through a study using the gold standard on the population at risk. Some of the individuals with no diagnosis during our study may be diagnosed after a longer time interval than we were able to follow‐up. The detection of precancerous lesions is also very useful clinically as it enables prevention of future cancer and this outcome was not included in this study.

### Comparison with other literature

Our analysis was designed to validate independently the results obtained by *Kinar* et al. Following their method, we have reported values for risk score cut offs corresponding to a sensitivity of 50% (≥83.47) and for a specificity of 99.5% (≥99.78). *Kinar* et al. undertook further analyses based on stage of cancer diagnoses. These were not available for our analyses in CPRD but we intend to carry out further analyses through linkage to the Cancer Registry. *Kinar* et al. derived measures of absolute risk for patients identified, but for our study these were not available. We attempted this through a sensitivity analysis and derived an estimate of the PPV of 8.8% for the high‐risk score cut off. However, identifying a meaningful “cohort” population in retrospective studies such as these is problematic.

Comparing our results with those of Hippisley‐Cox and Coupland 10, a study independently validated by Collins and Altman 12, we have derived comparable (although slightly lower) measures of discrimination for the Israeli algorithm. The Hippisley‐Cox and Coupland study included a number of predictive factors including symptoms recorded in primary care electronic records. A record of anemia was also included, but not FBC indices. We are unable to confirm through this present study our underlying premise that the Israeli risk score can identify cases at an earlier cancer stage than approaches based on symptoms. However, in terms of absolute risk, our estimated PPV for the highest risk groups (8.8%) is higher than the observed risk associated with Hippisley‐Cox and Coupland's upper 1% of predicted risk (5.2%).

### Clinical interpretation

Application of the Medial algorithm to routinely collected primary care data offers a potential additional means of identifying those at risk of colorectal cancer. For a patient with a risk score value associated with 99.5% specificity, we estimate a risk of being diagnosed over the next 2 years of 8.8%. This outcome does not include additional patients who have a precursor lesion (adenoma), who will also benefit from early recognition.

As expected, there is a trade‐off between algorithmic performance (increasing as the interval to diagnosis reduces) and opportunity to intervene (increasing as the interval increases). Confirming the diagnosis in an individual found to be at risk usually involves invasive procedures such as colonoscopy, which is not itself without risk although serious complications are rare 16. The PPV value we have derived for the high‐risk score values is significantly higher than the 3% recommended by the National Institute for Health and Care Excellence as a threshold for fast‐track cancer referrals in the UK 17. However, given the limitations in deriving measures of absolute risk in retrospective cohort studies, this finding needs to be confirmed prospectively through further research. It is also unclear whether the individuals identified with the high scores already have symptoms justifying investigation, or whether these patients are indeed being identified at an earlier, perhaps presymptomatic phase in the cancer process. Even where those identified are already symptomatic, their identification through use of the algorithm might still bring the (already justified) investigations forward. Failure to recognize and refer symptomatic individuals to exclude bowel cancer is part of the problem of late detection 18.

These findings may provide a role for the risk score as a tool to assist case finding in a range of settings. The score could be applied to primary care electronic health records and be automatically updated each time a FBC result appeared in the record. Patients with high (or increasing) values could be identified and considered for investigation. However, this high specificity approach will inevitably be limited in its ability to capture cases. At the less extreme cut off values, where sensitivity is higher, the algorithm could also help target those who do not take up the invitations for FOBt or who refuse colonoscopy when offered through the screening program.

Our results need to be followed up through further research investigating the utility of the risk algorithm embedded in the routine care setting, to optimize the early detection of colorectal cancer and improve health outcomes. This would involve measuring the risk scores of a greater proportion of the at risk population, and investigating pragmatically the additional benefit offered to early detection of bowel cancer.

## Ethical Approval

This is granted for all CPRD studies approved by the Independent Scientific Advisory Committee. No further study‐specific approval is required.

## Conflict of Interest

None declared.

## Supporting information


**Table S1.** Percentage of missing values for each blood level in the dataset of full blood counts retrieved from CPRD.
