# Supplementary material for: Evaluation of a prediction model for colorectal cancer: retrospective analysis of 2.5 million patient records
Source: Cancer Med. 2017 Sep 21;6(10):2453–60. doi: 10.1002/cam4.1183 (PMC5633543; doi:10.1002/cam4.1183)
Supplement: Supplementary file 1 — Table S1. Percentage of missing values for each blood level in the dataset of full blood counts retrieved from CPRD. [file CAM4-6-2453-s001.docx]

**Appendix**: Percentage of missing values for each blood level in the data set of full blood counts retrieved from CPRD.

| **Blood index** | **% of missing values** |
| --- | --- |
| Red blood cell count | 8.3 |
| White blood cell count | 8.4 |
| Mean platelet volume | 86.6 |
| Haemoglobin | 5.0 |
| Haematocrit | 11.6 |
| Mean corpuscular volume | 9.4 |
| Mean corpuscular haemoglobin | 8.4 |
| Mean corpuscular haemoglobin concentration | 11.5 |
| Red blood cell distribution width | 97.6 |
| Platelets | 9.1 |
| Eosinophils | 16.4 |
| Eosinophils (%) | 16.3 |
| Neutrophils | 14.5 |
| Neutrophils (%) | 13.5 |
| Monocytes | 15.9 |
| Monocytes (%) | 14.6 |
| Basophils | 22.2 |
| Basophils (%) | 21.0 |
| Lymphocytes | 14.2 |
| Lymphocytes (%) | 14.2 |
